# Beyond adult speech: why research on language development should consider speech from children

**DOI:** 10.1093/cdpers/aadaf004

**Published:** 2026-01-07

**Authors:** Federica Bulgarelli

**Affiliations:** Department of Psychology and Department of Learning and Instruction, University at Buffalo—SUNY, Buffalo, NY, United States

**Keywords:** child-produced speech, language development, siblings, early childhood education, peer language

## Abstract

Children’s language development is tied to their linguistic input. While most of this research has focused on adult speech, around the world, children regularly engage with other children, in the home or in early childhood education settings. Here, I contextualize the frequency of interactions with other children. I then highlight properties of speech from children, such as acoustic signature and content, which might make other children’s speech particularly relevant for language learning. Finally, I discuss how considering speech from children allows scholars to create theories that include societies around the world, where caretaking often involves more children and higher birthrates are common. I conclude that theories of language development should consider speech from other children as an important component.

Children’s language development is inherently tied to their linguistic input, but exactly how children’s experiences relate to language outcomes remains unknown. For example, how many words infants hear and how diverse those words are predict language development (e.g., Hart & Risley, 1995; Huttenlocher et al., 2010; Rowe et al., 2012). This research has focused almost exclusively on speech from adult caregivers. Hart and Risley’s (1995) seminal study calculated quantity estimates for speech by just one primary caregiver. Similarly, the commonly used Language ENvironment Analysis Software (LENA; Greenwood et al., 2011) calculates quantity estimates as adult word counts, and studies using LENA to measure linguistic input often report quantity estimates only for adult speech (see Wang et al., 2020).

Despite decades of research linking adult input to children’s language development, children do not interact exclusively with adults and development happens in contexts that can include other interlocutors. Since children’s experience with different communicative partners shapes their language use (Rowe & Weisleder, 2020), the focus on adult input does not fully capture children’s linguistic experiences and provides an incomplete picture of how language is learned. Ethnographic studies show that, in 40% of societies, during infancy, people besides mothers have an important caretaking role (Barry & Paxson, 1971), and in many cultures, children spend most of their days with other children (Scaff et al., 2024). In this article, I highlight the importance of considering speech from other children as an integral source of input for language development, focusing on two contexts: the home and early education settings. I also emphasize the likely prevalence of speech from children and the reasons it could influence learning.

## The frequency of interactions with other children

Around the world, children tend to live with other children (see Table 1 for the average number of individuals under age 20 in households worldwide; United Nations, Department of Economic and Social Affairs, Population Division, 2019). In Bolivia, Tsimane children have 2.9 siblings on average (Scaff et al., 2024). In The Gambia, infants hear language input from as many as seven siblings and eight nonsibling children (Katus et al., 2024). The experience of spending time with other children is also seen in societies with lower levels of fertility: Among French mothers and children (Heude et al., 2016), 2–3 year olds had older siblings who were on average 3.7 years older, and 47% of 2–3 year olds had a younger sibling by the time they were 6 (Havron et al., 2019). Similarly, 60% of 8- to 36-month-old Norwegian children had one or more older siblings, and 10% had one or more younger siblings, with an average gap of 3.6 years (Simonsen et al., 2014). In a Singaporean study, the average sibling age gap was 49 months (Havron et al., 2022). In the United States, 80% of children live in a household with other children (Current Population Survey, 2022). These examples highlight that around the world, young children often have at least one younger or older sibling within 5 years of their age.

Children also interact with other children in child care settings. In the United States, 54% of 3–4 year olds are reportedly in child care (Current Population Survey, 2022; de Brey et al., 2021). In Europe, 93% of children aged 3 years or older were enrolled in early childhood education in 2022 (Eurostat, 2024). While the number of children in a classroom varies, a typical U.S. preschool classroom includes 20–25 children and two teachers (Duncan et al., 2020). For younger children, the National Association for the Education of Young Children (NAEYC) recommends that classrooms have a maximum of 12 toddlers (American Academy of Pediatrics, American Public Health Association, & National Resource Center for Health and Safety in Child Care and Early Education, 2019; NAEYC, 2018). In Germany, the number of children under 3 years old in a classroom ranges from 8 to 15, and the number of 3–6 year olds in a classroom ranges from 20 to 25 children (Eurydice, 2024). Similarly, in Asian countries (e.g., China, Indonesia), the maximum class size for 2–3 year olds is 14–20 children, with teacher–child ratios of 1:8 (Women, Business and the Law, 2022). These data suggest that children around the world spend time in early childhood classrooms, where they may interact with dozens of other children.

Children’s interactions include frequent speech from other children. In one study, half of Mayan children’s input and 10% of North American children’s input came from other children (Shneidman & Goldin-Meadow, 2012; see also Galindo & Montag, 2025). Similarly, in a study in Lesotho, more than half of children’s input came from other children, compared to 8.5% of speech for age-matched French children (Loukatou et al., 2022). In a Tsimane forager–horticulturalist community, 38% of directed input to children younger than 3 was provided by siblings younger than 12 (Cristia et al., 2019). In a recent analysis of daylong recordings in North America (Warlaumont et al., 2024), 3- to 6-month-old children with older siblings heard speech from other children once every minute, on average (Bulgarelli, 2023).

In child care settings, while children younger than 1 do not provide consistent language input, toddlers and preschoolers undoubtedly receive linguistic input from their peers. Specifically, preschoolers spent similar amounts of time talking to peers and adults (Blackstock-Bernstein, 2019), and spent approximately 52% of their time in group settings with other children and 33% in whole-class activities (Justice et al., 2022). Thus, speech from children is a common feature of language input, and in some societies and contexts, it is as frequent as speech from adults.

## The influence of other children on language development

**Figure 1:**
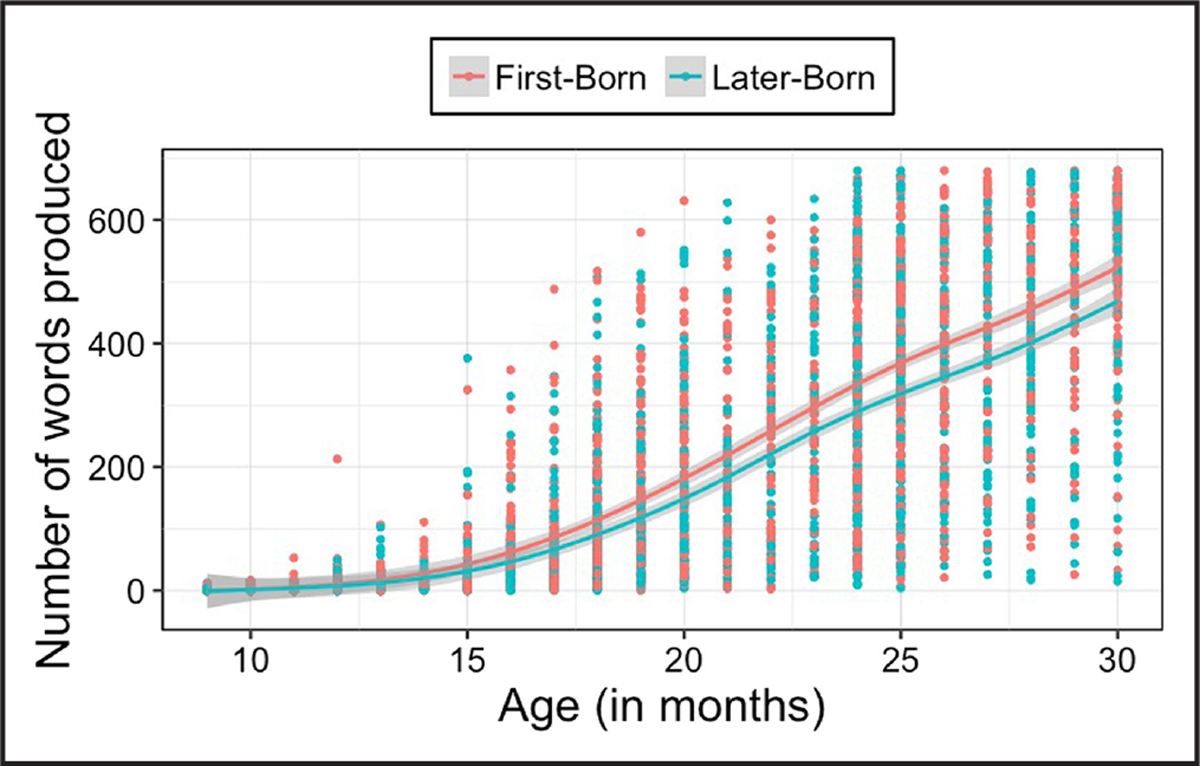
Productive vocabulary for 9- to 30-month-old first-born and later-born children. First-born children are in pink and later-born children are in blue. This graph combines data from words and gestures (9–18 months) and words and sentences (16–30 months). Data source: Wordbank (Frank et al., 2017).

Research suggests that frequent experiences with children can negatively influence language development. Specifically, infants with older siblings have smaller vocabularies (Pine, 1995), reach the 50-word milestone later (Bornstein et al., 2004), and have weaker language skills than eldest children (Havron et al., 2019; see also Havron et al., 2022). This pattern is also evident if we analyze data from children learning American English in Wordbank, an open database of children’s vocabulary development (Frank et al., 2017); differences emerged at 14 months and continued until 30 months (see Figure 1; Hippe & Ferjan Ramírez, 2022; Rosslund et al., 2025). Thus, early vocabularies, which predict later language skills (Marchman & Fernald, 2008), are reduced for younger siblings.

In contrast, in other research, interacting with siblings or peers with stronger language skills supports language development. In one study, 3-year-old children who had older siblings with more advanced language skills also had larger vocabularies (Prime et al., 2014). In education contexts, 4-year-old children had stronger receptive and expressive language skills when their classmates’ language skills were stronger (Mashburn et al., 2009), and children who vocalized more with their peers also had larger vocabularies (Perry et al., 2022). More generally, academic achievement for children is positively associated with their peers’ skills (Foster, 2021; Henry & Rickman, 2007).

These findings imply that interacting with siblings and classmates influences language development. However, whether these interactions are beneficial or pose a challenge, and the mechanism through which these effects occur, remains unclear. Some hypothesize that potential negative effects are related to differences in adult (directed) language input for first-born relative to later-born children (Blake, 1981; Galindo & Montag, 2025; Jones & Adamson, 1984; Laing & Bergelson, 2024; McCartney et al., 1991) and that occur when child–teacher ratios are higher (Schaffer & Liddell, 1984).

However, adult speech does not fully capture variability in language development since meta-analyses report correlations between adult input and language outcomes ranging from *r* = .30 for in-home contexts (Anderson et al., 2021) to *r* = .11 for school contexts (Jiang et al., 2025). To that end, considering speech from people other than mothers improves predictions of children’s vocabulary (Shneidman et al., 2013, 2016; cf. Shneidman & Goldin-Meadow, 2012), and infants with larger social networks (i.e., more interactions with individuals besides their primary caregivers) also have larger vocabularies (Okocha et al., 2024). Beyond language, children’s experiences with more diverse social contexts (e.g., linguistically and racially diverse neighborhoods) also influence social learning and preferences (Howard et al., 2014; Immel & Liberman, 2024). In short, children’s varied experiences with other people shape from whom and what they learn.

## How speech from children may change the learning process and outcomes

Next, I address how experiences with other children could shape language development. I show that children may find speech from other children more interesting to attend to and that speech from children features different regularities, but may be more difficult to comprehend. I also show that while later-born children may have smaller vocabularies (see Figure 1), they learn certain words earlier than first-born children. Given the prevalence of siblings and peers in children’s environments worldwide, these findings highlight the need to directly investigate the role of speech from other children in language development.

**Figure 2:**
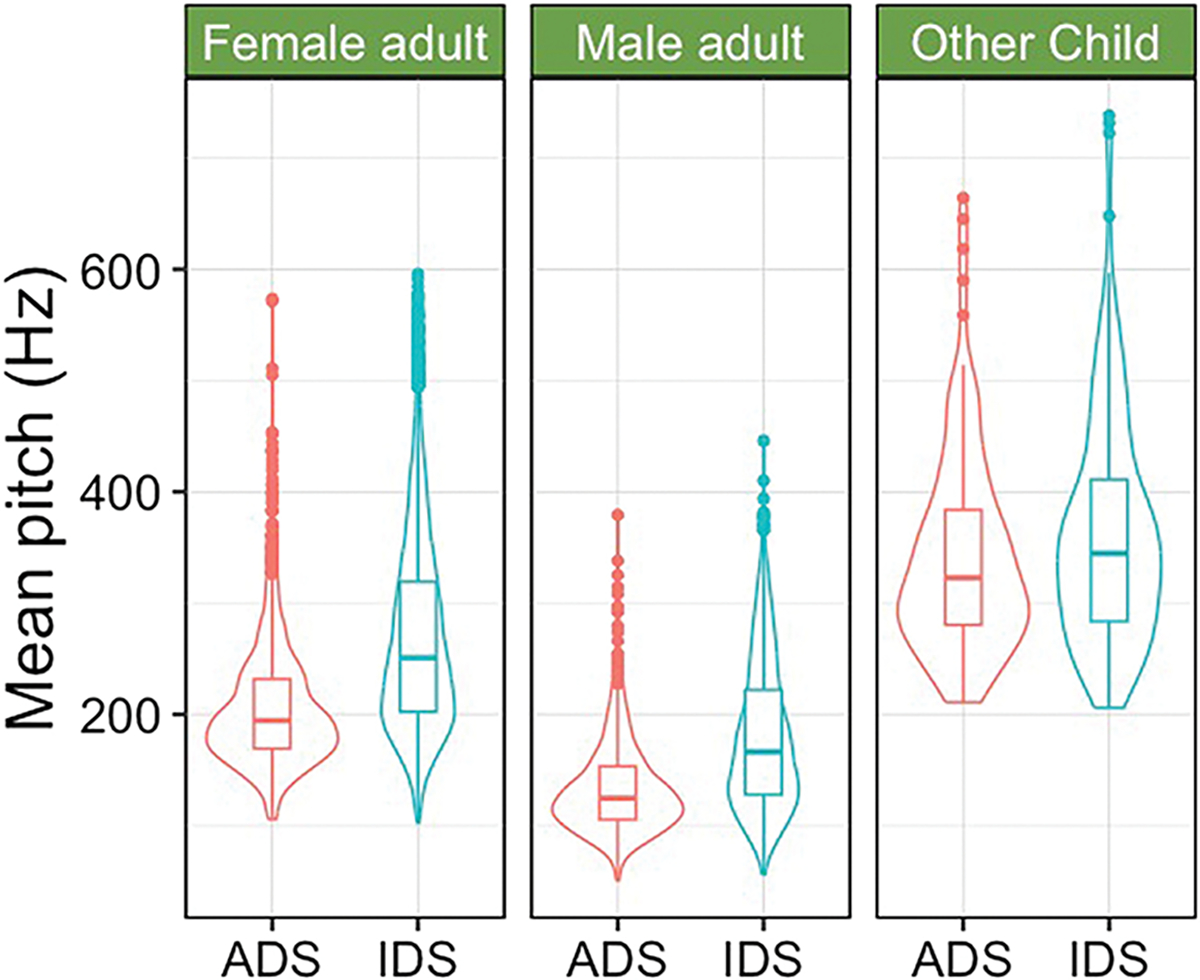
Mean pitch of highly frequent words produced by female and male adults and other children. The right side of each panel represents words in infant-directed speech (IDS); the left side of each panel represent words in adult-directed speech (ADS). Data sources: Bulgarelli et al. (2021) and Bulgarelli and Bergelson (2024).

Infant-directed speech (IDS) (also called child-directed speech) is a speaking style caregivers can engage in that features higher pitch, more variation in pitch, and longer duration (Fernald et al., 1989). While not universal (Casillas et al., 2020), infants around the world prefer listening to IDS over adult-directed speech (ADS) (ManyBabies Consortium, 2020). Speech from children shares many properties with IDS (Lee et al., 1999; Tingley & Allen, 1975). For example, common nouns produced by other children (e.g., baby, ball; see Bulgarelli et al., 2021) are longer in duration and higher in mean pitch, even compared to words produced by adults in IDS (see Figure 2; Bulgarelli & Bergelson, 2024). Additionally, preschoolers modify their speech when speaking to infants (Loukatou et al., 2022; Sachs & Devin, 1976; Shatz & Gelman, 1973). Thus, the similarities between speech from children and IDS (whether spontaneous or adjusted in interactions with infants) might make speech from children particularly interesting. In fact, in a recent study, 8–20-month-old children from Peru and Switzerland preferred listening to surrounding (i.e., not infant-directed) speech from children than to speech from adults, regardless of language familiarity, and infants listened similarly to surrounding speech from children and adult IDS (Schick et al., 2025).

Since infants do attend to speech from other children and this kind of speech occurs quite frequently, we cannot assume that children are learning only from the regularities and properties of adult speech. I examined recordings with preschoolers (since preschool-aged children span the average age of 1-year-olds’ older siblings) from the CHIld Language Data Exchange System (CHILDES; MacWhinney, 2000) and speech from siblings and other children from the Study of Environmental Effects on Developing LINGuistic Skills (SEEDLingS; Kalenkovich et al., 2025), which included siblings and other children: Half of children’s (i.e. siblings and preschoolers) most frequent nouns differed from parents’, and more than 30% of the nouns differed when combining frequency across children and adults (see Table 2). Thus, children’s speech differs from that of adults, and attending to speech from older children could change the regularities of the input.

The presence of other children is also thought to provide more and different opportunities to learn pronouns; children who hear a mix of pronouns and names for self- and other-referring words (e.g., “I, me, mine, Mommy”) learn to use pronouns earlier (Smiley et al., 2011). Environments with other children may include more third-person references to other people (Pine, 1995), as suggested by data from CHILDES (MacWhinney, 2000). In an analysis of mothers’ and preschoolers’ use of the words “mom, mommy, and mother,” preschoolers produced three times as many of these third-person utterances as did mothers in the same recordings and mothers of 8- to 18-month-old children. Since words heard more frequently tend to be produced earlier (Goodman et al., 2008; Swingley & Humphrey, 2018), we must consider that the regularities available in child-produced speech may differ from those available when attending only to adult-produced speech.

However, children with older siblings or in child care settings may also be attending to speech that could be harder to understand. Adults are less accurate at transcribing speech from children, but they improve significantly for speech from 5.5 year olds compared to younger children (Hustad et al., 2021; Yu et al., 2023). Similarly, 2.5 year olds are less accurate at processing speech produced by same-age peers than by adults (Cooper et al., 2018), though toddlers’ processing improves when comprehending speech from 7 year olds (Bernier & White, 2019).

Evidence suggests that aspects of child-produced speech do shape language development. For example, while children hear less overall directed speech from other children than from adult women (Bunce et al., 2024), surrounding input (i.e., language that is overheard) from other children is a better predictor of children’s productions than surrounding input from adults or directed input from adults and children (Schick & Stoll, 2025). Furthermore, if we calculate age of acquisition (the age at which half of the children understand a word) for words from the words and gestures data from Wordbank for first-born and later-born children separately, we see that later-born children learned some words earlier than first-born children (see Table 3; see also Frank et al., 2017). Also, later-born children are thought to learn pronouns faster than first-born children because they overhear conversations between older siblings and parents (Oshima-Takane & Robbins, 2003; Pine, 1995). Thus, even if child-produced speech is overheard when it is directed to others, children may attend to it (Akhtar et al., 2001). Researchers should test whether words learned earlier by later-born children are also produced more frequently by older (e.g., preschool-aged) children.

## Moving forward

I have identified some ways that interacting with other children (siblings or peers) likely shapes children’s experiences. To understand language development in the context of other children, researchers need to collect new data. In CHILDES (MacWhinney, 2000), the largest collection of children’s language interactions, only 6% of children have recordings that include siblings, and 4% of recordings include a peer or an unrelated child (across all languages). Similarly, the Play and Learning Across a Year (PLAY) project, which aims to collect infant–parent interactions from more than 900 babies across the United States, asks that only mothers be present during recordings (Adolph et al., 2016). Furthermore, studies rarely report children’s sibling status or time in child care settings (if that information is collected at all; see Singh et al., 2024), making it challenging to investigate input or experimental results as a function of experience with other children.

To understand how the regularities of input change when speech from other children is considered and how experience with other children influences development, researchers need to collect data that answer those specific questions. These efforts could include play sessions with parents and multiple siblings (see Filipski et al., 2025), daylong recordings with larger families (and corresponding analyses; Galindo & Montag, 2025; Hippe & Ferjan Ramírez, 2022), or recordings in child care settings (e.g., Perry et al., 2018; Sparks et al., 2024).

In addition to measuring the frequency of speech from other children, researchers should consider children’s attention to overheard vs. directed speech from other children, and its relation to language development, given studies’ findings regarding mixed effects of overheard speech (Shneidman et al., 2013; Shneidman & Goldin-Meadow, 2012). Since technology does not yet allow for automated quantity estimates and transcriptions of speech from other children, transcribing and analyzing these data may be costly, but doing so is necessary to understand the experiences of most children around the world.

Researchers also need to collect controlled, in-laboratory experimental data that allow us to answer questions about how speech from children is processed and how experience with this speech shapes other learning mechanisms. For example, while some research suggests that speech from children is more challenging to understand than speech from adults, does experience with this speech reduce or eliminate the challenge? If infants attend to this speech more frequently and it is harder to comprehend, this could explain the reported differences in language development for younger siblings (e.g., Havron et al., 2019). However, difficulty processing child-produced speech could reflect a lack of experience since toddlers learn best when speakers match their type of experience (Fennell & Byers-Heinlein, 2014).

Researchers should also test more directly whether and how experience with child-produced speech influences language processing, as well as how it may result in more robust and flexible language acquisition, possibly in ways similar to how experiences with accented speech and acoustic variability shape word recognition (Van Heugten & Johnson, 2017), word learning (Bulgarelli & Bergelson, 2022, 2023), and production (Bulgarelli & Bergelson, 2024). Similarly, since infants and toddlers learn and remember new words from IDS better than from ADS (Graf Estes & Hurley, 2013; Ma et al., 2011), how does learning words from other children influence learning and retention across early childhood? More generally, researchers need to explore whether children can and do (choose to) learn from other children, and whether that learning is specific to certain types of information (e.g., information about toys vs. foods; VanderBorght & Jaswal, 2009).

Effects of speech from other children will undoubtedly vary as a function of the age of both the target child and the other children in the environment. Certain properties of language input are more relevant earlier or later in development; for example, repetition benefits younger infants, while linguistic diversity benefits older children (see Rowe & Snow, 2020; Schwab et al., 2018). Some research suggests that a smaller age gap between siblings supports language learning (Gurgand et al., 2023; Havron et al., 2022; but see Havron et al., 2019); researchers should test whether this finding is related to older siblings’ language at specific ages. For example, a 3 year old’s language might feature repetitions that makes it ideal for younger infants to learn from, while a 5–7 year old’s language might include more linguistic diversity than an infant can benefit from (e.g., Rowe & Snow, 2020). In preschool settings, where children are often with other children in a narrower age range, having peers with stronger language skills results in more language growth (Mashburn et al., 2009). Therefore, older siblings and peers may shape language development differently than same-aged peers.

## How considering the speech of other children is beneficial

Spending time with other children is the modal experience for children around the world, and children do learn from their peers, starting in infancy (Kuczynski et al., 1987; Ryalls et al., 2000; Shutts et al., 2010; VanderBorght & Jaswal, 2009). Given the importance of peer learning across social contexts and societies (see Lew-Levy et al., 2023), children likely also learn aspects of language from peers. Any theory of language development that does not consider speech from other children falls short of capturing a frequent source of input, limiting its generalizability.

Including speech from other children in theories of language development is critical as science is diversified. Table 1, which shows that there are more children in non-Western than Western households, highlights this point, as does research that shows that in all countries, children of parents without tertiary degrees have more siblings than children of parents with advanced degrees (Präg et al., 2020). Considering speech from children by default would result in theories of language development that account more readily for the variety of experiences around the world. For example, in some communities (e.g., Tseltal Mayan, Rossel Island Papuan), IDS from adults is infrequent and, thus, not a requirement for language development (Casillas et al., 2020, 2021). However, a theory of language development that includes speech from other children might find that children across communities similarly hear speech that features properties of IDS (from children or adults) and that these could serve similar functions.

Research also suggests that adult input in child care classrooms varies as a function of parents’ socioeconomic status. Specifically, in a U.S. study, receiving a child care subsidy correlated negatively with hearing adult words, taking part in conversational turns, and child vocalizations, even in classrooms highly rated based on state guidelines (Duncan et al., 2020). Even if teachers are talking less, children are talking, highlighting the importance of considering speech from children to fully capture the language environments of a diverse array of children.

## Conclusion

The research I have reviewed suggests that children around the world regularly interact with other children, whether in the same household or in classrooms, and that aspects of child-produced speech may shape processing and learning. Despite these findings, research has focused primarily on the role of adult language input in language development, neglecting speech from other children. The questions I have asked set the stage for understanding how speech from other children may influence multiple aspects of language learning from birth through the preschool years. Answering these questions will lead to more well-rounded and representative theories of language development that account for real-life experiences.

## Supplementary Material

SupplementalTable

Supplementary material is available at Child Development Perspectives online.

## Figures and Tables

**Table 1:** Average number of household members under age 20 across regions.

Region	Europe	North America	Eastern and South-Eastern Asia	Latin America and Caribbean	Northern Africa	Western Asia	Sub-Saharan Africa

**Average number of house-hold members under 20**	1.7	1.7	2.2	2.2	2.8	2.8	3.3

*Note*. Source: United Nations, Department of Economic and Social Affairs, Population Division (2019).

**Table 2:** Top 10 nouns produced by parents and children/siblings and combined.

	Parent		Children		Combined	

**CHILDES Children are 3–5.5 years old**	1	Car	1	Car	1	Car
	2	Book	2	**D**og	2	Book
	3	Bed	3	Bed	3	Bed
	4	Cat	4	Cat	4	**Dog**
	5	Table	5	Door	5	Cat
	6	Picture	6	Train	6	**Door**
	7	Head	7	Milk	7	Head
	8	Box	8	Book	8	**Milk**
	9	Room	9	Head	9	**Train**
	10	Door	10	Elephant	10	Table
**SEEDLingS Children are any sibling or other child in the recordings**	1	Book	1	**Baby**	1	Book
	2	Baby	2	Book	2	Baby
	3	Hand	3	**Water**	3	Hand
	4	Dog	4	Hand	4	Dog
	5	Diaper	5	**Car**	5	**Ball**
	6	Ball	6	Ball	6	Diaper
	7	Foot	7	Dog	7	**Water**
	8	Water	8	**Cat**	8	Foot
	9	Bottle	9	**Apple**	9	Bottle
	10	Mouth	10	**Hair**	10	**Car**

*Note*. For CHILDES (CHIld Language Data Exchange System), I analyzed speech from parents (mother or father), preschoolers (target child), or both for English-speaking target children aged 3–5.5 years; words were limited to nouns on the words and sentences form of the Communicative Development Inventory. In SEEDLingS (Study of Environmental Effects of Developing LINGuisic Skills), I analyzed words produced by parents (mother and father), siblings and other children, or both; for recordings that included siblings or other children, any coded noun was included. Bolded words represent words that have a higher ranking for preschooler and combined frequency relative to parent frequency, highlighting that children’s most frequent words differ from those of adult caregivers. Considering speech from children (older siblings or peers) could change the regularities of the input.

**Table 3:** Examples of words that have an age of acquisition earlier for first-born children (first-born first), later-born children (later-born first), or are even.

		First-born first	Even	Later-born first

**Understands**	Number	59	137	48
	Examples	Meow, quack quack, bird, bunny, truck, flower, tree	Cat, dog, train balloon, cheerios, cracker, hat, mouth	Ball, doll, cookie, potty, blanket, slide, ride, me, you

*Note*. The age of acquisition is the age at which 50% of children understand a word.

Source: Words and gestures form (Wordbank; Frank et al., 2017).

## Data Availability

All code for analysis using openly available data presented in this article is available on OSF: https://osf.io/9w7m6
