# Supplementary material for: Beyond adult speech: why research on language development should consider speech from children
Source: Child Dev Perspect. 2026 Jan 7;20(1):9–17. doi: 10.1093/cdpers/aadaf004 (PMC12959929; doi:10.1093/cdpers/aadaf004)
Supplement: aadaf004_Supplementary_Data [file aadaf004_supplementary_data.docx]

**Supplemental Table 1**

*Sociodemographic Characteristics of Studies Cited in the Article, Alphabetized by First Author’s Last Name*

| **Study** | **Country of Participants** | **Sex** | **Race/Ethnicity** | **Socioeconomic Status** | **Age** | **Ratio/Siblings** |
| --- | --- | --- | --- | --- | --- | --- |
| Ahkar et al., (2001) | United States | N = 88  Study 1: 13 F, 11 M  Study 2: 16 F, 16 M  Study 3: 18 F, 14 M | Not reported | Middle income | Study 1: 2;6  Study 2: 2;1  Study 3: 2;1 |  |
| Bernier & White (2019) | Canada | N = 88  Study 1: 48  Study 2: 40 | Not reported | Not reported | Study 1: 1;10  Study 2: 1;10 | Study 1: 23 had older siblings  Study 2: 10 had older siblings |
| Blackstock-Bernstein (2019) | United States | N = 117 children  56 F, 61 M  N = 61 teachers  61 F | Children:  94% Hispanic  Teachers:  84% White | Children:  90% lived in households receiving public assistance or had incomes <130% of the federal poverty level  Teachers:  39 BA, 24 Associate’s, 29 some college, 6 high school or unspecified | Children: 4;6 |  |
| Bornstein et al., (2004) | United States | N = 110  55 first-borns (26 F, 29 M)  55 second-borns (26 F, 29 M) | Not reported | Lower-middle class to upper middle class  Hollingshead index:  First-borns = 53.95  Second-borns = 55.11 | First-borns at 1;8  Second-borns at 1;8 | Average age gap of 2;11 |
| Bulgarelli & Bergelson (2022) | United States | N = 126  Study 1a N = 54  26 F, 28 M  Study 1b N = 18  11 F, 7 M  Study 2 N = 54  28 F, 26 M | Study 1a:  74% White  4% Black or African American  20% other or multiracial  Study 1b:  100% White  Study 2:  89% White  3% Asian  8% other or multiracial | Study 1a:  24 advanced degree  24 associate’s or bachelor’s  Remaining < associate’s or bachelor’s  Study 1b:  9 advanced degree  7 associate’s or bachelor’s  2 < associate’s or bachelor’s  Study 2:  35 advanced degree  14 bachelor’s or associate’s  2 < bachelor’s or associate’s | 0;7-0;9 |  |
| Bulgarelli & Bergelson (2023) | United states | N = 54  34 F, 20 M | 87% White  13% Multiracial, multiethnic, other | 32 advanced degree  16 associate’s or bachelor’s  2 < associate’s or bachelor’s  4 did not report | 1;1-1;3 |  |
| *Bulgarelli & Bergelson (2024) | United States | N = 44  21 F, 23 M | 42 White  2 multiracial | 18 mothers had an associate’s or bachelor’s degree, 22 mothers had an advanced degree | Longitudinal from 0;6-1;5 |  |
| *Bulgarelli et al., (2021) | United States | N = 44  21 F, 23 M | 42 White  2 multiracial | 18 mothers had an associate’s or bachelor’s degree, 22 mothers had an advanced degree | Longitudinal from 0;6-1;5 |  |
| Bunche et al (2024) | United States, Canada, England, Argentina, Mexico, Papa New Guinea | N = 69 total  34 M | Not reported | Not reported | 1;2 (0;2-3;0) | Household size:  5.1 (2-14) |
| Casillas et al., (2020) | Mexico | N = 10  5 F, 5 M | Not reported | 2 No education  2 Preparatory  3 Primary  3 Secondary | 0;2 to 3;0 | Mean household size: 8 |
| Casillas et al., (2021) | Papua New Guinea | N = 10  5 F, 5 M | Not reported | 1 Preparatory  4 Primary  5 Secondary | 0;1 to 2;11 | Mean household size: 7 |
| ManyBabies Consortium | Around the world | Not reported | Not reported | Not reported | 0;3 – 1;3 |  |
| Cooper et al., (2018) | Canada | N = 54 | Not reported | Not reported | 2;6-3;0 |  |
| Cristia et al., (2019) | Tsimane, Bolivia | Not reported | Not reported | Forager-farmer community | 0-1;0 | Total fertility rate of 9 births, 30 month interbirth interval |
| Duncan et al., (2020) | United States | N= 44 59% M | 45% White  25% African American  18% Hispanic  11% Other | 84% received child-care subsidy (low income) | 4;9 | 2 teachers per 20-25 children |
| Fennel & Byers-Heinlein (2014) | Canada | N = 61  Bilinguals N = 30  16 F  Monolingual N = 31  15 F | Not reported | Not reported | Bilinguals:  1;5 (1;4-1;6)  Monolinguals:  1:5 (1;4-1;6) |  |
| Fernald et al. (1989) | French, Italian, German, Japanese, British English and American English | N = 60  30 F, 30 M | Not reported | Not reported | 0;10-1;2 |  |
| Foster (2021) | United States | N = 357  49% F, 51% M | 29% Black  24% White  42% Hispanic | Low-income | 4;6 |  |
| Galindo & Montag (under review) | United States | N = 26  15 F, 11 M | Not reported | Not reported | 0;6-1;0 | N=13 with older siblings |
| Graf Estes & Hurley (2013) | United States | N = 82  Study 1 N = 28  13 F  Study 2 N = 26  13 F, 13 M  Study 3 N = 28  14 F | Not reported | Not reported | 1;5.5 (1;5-1;6) |  |
| Greenwood et al., (2011) | United States | N = 30 | 77% European American  10% African American  7% Hispanic  Remainder Asian, Pacific-Islander, multi-race | Middle to Upper SES | Longitudinal,  1;3 at start |  |
| Gurgand et al., (2023) | France | N = 12296  49% F | Not reported | Average education: 3.85 (3 = high school diploma, 4 = 2 years after high school diploma) | 2;0 | 5457 only children  4683 had 1 sibling  1727 had 2 siblings  429 > 2 siblings |
| Hart & Risley (1995) | United States | N = 42 | Not reported | Low to high SES | Longitudinal, 0;7-3;0 |  |
| Havron et al., (2019) | France | N = 1276 | Not reported | Not reported | 2;0, 3;0, 5;0-6;0 | 43% had older sibling  Average age gap = 3;8 |
| Havron et al., (2022) | Singapore | N = 677  47.71% F | 61% Chinese  20% Malay  19% Indian | Not reported | 2;0, 4;0, 4;6 |  |
| Henry & Rickman (2007) | United States | N = 630  52.8% M | 50.4% White  38% African American  11.6% Other | 24% less than high school education  27.2% high school diploma  48.8% more than high school | 4 year olds | 39% had an older sibling  Average age gap = 4;1 |
| Hippe & Ferjan Ramirez (2022) | United States | N = 24  12 M | Not reported | Hollingshead Index = 48.8  Range of SES | Longitudinal,  0;6, 0;10, 1;2, 1;6, 2;0 | 38% had older sibling |
| Howard et al., (2014) | United States | N = 82  Between Subjects N = 50  27 F, 23 M  Within Subjects N = 32  18 F, 14 M | Not reported | Between Subjects:  Mean Family Income $91k  Within Subjects:  Mean Family Income $71k | 1;7 |  |
| Hustad et al., (2021) | United States | N = 538  281 F, 257 M | 471 White  7 Black  9 Asian  1 American Indian  1 Native Hawaiian/Pacific Islander  31 More than 1 race | 2-Factor Hollinghead Social Index Mean = 55.55 | 2;6 to 10;0 |  |
| Huttenlocher et al., (2010) | United States | N = 47 | 9 African American  3 Asian  5 Hispanic  30 White | 5 High School only  10 Some college  16 Bachelor’s degree  16 Advanced degree | Longitudinal,  1;2 to 3;10 | 29 first-borns  18 later-borns |
| Immel & Liberman (2024) | United States | N = 96  53 F, 43 M | 64 White  15 Multiracial  10 Hispanic/Latino  2 Asian  1 White | 87.5% had one or more parents with a college degree | 0;9 (0;8-1;2) |  |
| Jones & Adamson (1984) | United States | N = 32  16 F, 16 M | Not reported | Middle class | 1;8 | 16 first-borns  16 second-borns  Mean age gap = 2;9 |
| Justice et al., (2022) | United States | N = 1095  49% F | 67% White  8% Black  12% Hispanic/Latino | 45% with college degree or higher |  |  |
| Kalenkovich et al., (2025)* | United States | N = 44  21 F, 23 M | 42 White  2 multiracial | 18 mothers had an associate’s or bachelor’s degree, 22 mothers had an advanced degree | Longitudinal from 0;6-1;5 |  |
| Katus et al., (2024)  **Gambia** | Gambia | N = 200  No differences in sex ratio | Mandinka ethnic group | 59% no formal education  13% limited formal education  3% complete primary education  18% some secondary education  6% complete secondary education | Longitudinal,  0;5, 0;8, 1;0, 1;6, 2;0 |  |
| Katus et al., (2025)  **UK** | United Kingdom | N = 60  No differences in sex ratio | Not reported | Majority received undergraduate or postgraduate education | Longitudinal,  0;5, 0;8, 1;0, 1;6, 2;0 |  |
| Kuczynski et al., 1987 | United States | N = 24  13 F, 11 M | Mothers were White | Mothers were middle class, high school or college graduates | Longitudinal,  Younger: Mean 1;3 in the middle  Older:  Mean 2;5 in the iddle | N = 16 had younger siblings |
| *Laing & Bergelson (2024) | United States | N = 44  21 F, 23 M | 42 White  2 multiracial | 18 mothers had an associate’s or bachelor’s degree, 22 mothers had an advanced degree | Longitudinal from 0;6-1;5 | Mean age gap: 4;1 |
| Lee et al., (1999) | United States | N = 436 children  207 F, 229 M  N = 56 adults  27 F, 29 M | Not reported | Not reported | 5;0-18;0 |  |
| Loukatou et al., (2022)  **France** | France | N = 3  2 F, 1 M | Not reported | Not reported | Longitudinal,  1;0-3;0 | 1 had one older sister  1 had two older sisters  1 had two older brothers |
| Loukatou et al., (2022)  **Africa** | Lesotho, Africa | N = 3  2 F, 1 M | Not reported | Not reported | Longitudinal, from 2;1 to 3;2 | 1 had older sibling  2 had older cousins in the household |
| Ma et al., (2011) | United States | N = 64  Study 1 N = 48  24 F  Study 2 N = 16  9 F |  |  | Study 1: 1;9 (1:8-1:10)  Study 2: 2;4 (2;2-2;5) |  |
| Marchman & Fernald (2008) | United States | N = 28 | Primarily Caucasian | Primarily college-educated | Longitudinal,  2;1 and 8;5 |  |
| Mashburn et al., (2009) | United States | N = 1812  924 F, 888 M | 407 African American  196 Latino  923 White  262 Other  24 Missing | Average 13.1 years of maternal education | Preschool children | Average class size 17.6 kids  Average child-teacher ratio 7.67:1 |
| Okocha et al., (2024) | United States | N = 369  189 F, 177 M | 63% White  9% Black  6% Latinx  4% Asian  18% more than one | 44% annual income < 50k  Range from <25k to > 200,000k | 2;0 (1;0-3;1) |  |
| Oshima-Takane & Robbins (2003) | Canada | N = 14 |  |  | 1;9 | All later-borns |
| Perry et al., (2022) | United States | N = 29  17 F, 12 M | 13 White Hispanic  1 Mutiracial Hispanic  8 White  5 Black  2 Asian | Not reported | 3;0 |  |
| Pine (1995) | United States | N = 18  8 F, 10 M | Not reported | Middle class | Longitudinal, starting when first born was 0;11 | 9 first-borns  9 second-borns  Mean age gap 2;6 |
| Prime et al., (2014) | Canada | N = 385  50% M | 57% Caucasian  7% Black  27% Asian  9% other | Maternal education mean: 15.59  Household income: $65k-75k | 3;0 | 60% had 1 sibling2  40% had 2 or more |
| Rosslund et al., (2025) | Norway | N = 6163  51.4% F | Not reported | Median SES = lower-level university degree | 1;10 average  Range from 0;8 to 3;0 | 59.9% had one or more older siblings (range = 0-6)  10.3% had one or more younger siblings (range = 0-2)  Average age gap was 5.25 years |
| Rowe et al., (2012) | United States | N = 62  30 F, 32 M | 12 Black  35 White  6 Two or more races  9 Hispanic | Annual income:  5 Less than $15000  13 $15000-34999  8 35000-49999  12 50000-74999  10 75000-99999  14 100000 or more | Longitudinal, every 4 months from 1;2 to 3;10 |  |
| Ryalls et al., (2000) | United States | N = 30  16 F, 14 M | Majority Caucasian | Majority middle class | 1;4 (range 1;2 to 1;6) |  |
| Sachs & Devin (1976) | United States | N = 4 | Not reported | Not reported | 3;9 to 5;5 |  |
| Scaff et al., (2024) | Bolivia | N = 24  8 F, 16 M | Not reported | Not reported | 2;6 (range 0;6 to 4;10) | Average household size: 8  Average number of siblings: 2.9 |
| Schaffer & Liddell (1984) | United Kingdom | N = 16 teachers | Not reported | Not reported | Observed children were 2;7 |  |
| Schick, Daum, & Stoll (2025) | Peru, Switzerland | N =  Study 1 N = 127  N=67 in Peru, 37F  N = 60 in Switzerland, 29 F  Study 2 N = 61  28 F | Not reported | Not reported | Study 1: 1;1 (0;8-1;8)  Study 2: 1;2 (0;8-1;8) | Study 1 Peru: 3.85 siblings (range 0-10)  Study 1 Switzerland: 0.47 (range 0-2)  Study 2: 0.44 (range 0-4) |
| Schick & Stoll (2025) | Peru | N = 12  8 F, 4 M | Not reported | Not reported | 1;0 to 4;9 |  |
| Schwab et al., (2018) | United States | N = 41  22 F, 19 M | 100% African American fathers | Fathers’ M education: 12.5 (10-16) – high school degree | 2;0  Fathers’ M = 29y (18-52) |  |
| Shatz & Gelman (1973) | United States | N = 21  Study 1: 16  Study 2: 5  3 F, 2 M | Not reported | Middle to upper class | 4;4 (range 3;1 to 5;0) |  |
| Shneidman & Goldin-Meadow (2012) | Mexico  United States | N = 9 each  Mexico: 6 F, 3 M  USA: 7 F, 2 M | USA:  4 Caucasian  3 African American  1 Mixed Race  1 not reported | USA:  4 < 35,000  1 between 50,000-74999  4 > 75000 | 1;2 | Average number of children present  Mexico: 4.7  USA: 1.3 |
| Shneidman et al., (2013) | United States | N = 30  18 F, 12 M | Not reported | 5 graduated high school  6 some college  10 college degree  9 advanced degree | Longitudinal, every four months from 1;2 to 3;6 | 10 participants had siblings, 2.3 siblings on average |
| Shneidman et al., (2016) | United States  Mexico | N = 142  Study 1:  N = 76 from US  Study 2:  N = 66  36 Yucatec Mayan, 30 from US | Study 2:  US – Primarily White | Study 1: Primarily Middle class  Study 2: US Primarily Middle Class, 17.2 years of education  Mayan – 5.6 years of education | Study 1: 2;6  Study 2: 2;7 | Study 2:  Mayan children larger household size (2.6 siblings) than US (0.5 siblings) |
| Shutts et al., (2010) | United States | N = 64  22 F | 100% White | Not reported | 3;5 |  |
| Simonsen et al., (2014) | Norway | N = 6574 reports  51% F, 49% M | Not reported | 3% Basic education (<9 years)  30% Short further education (12 years)  42% Medium further education (14-16 years) 24% Long further education (>16 years) | 0;8 to 3;0 |  |
| Sparks et al., (2024) | United States | N = 26  50% male/female | 7.29% African American/Black  19.2% Asian American/Pacific Islander  38.5% Caucasian/White  7.69% Hispanic/Latinx  30.8% multiracial  3.85% other | Not reported | 2;11-5;11,  Mean = 4;6 |  |
| Tingley & Allen (1975) | United States | N = 20 | Not reported | Not reported | 5;0-11;5 |  |
| Van Heugten & Johnson (2017) | Canada | N = 80  Study 1 N = 40  21 M  Study 2 N = 40  23 M | Not reported | Study 1: 5.7*  Study 2a:6.1  Study 2b: 5.6  *measured on scale of 1 (some high school) to 7 (postgraduate degree) | Study 1: 1;0  Study 2a: 1;2  Study 2b: 1;6 |  |
| VanderBorght & Jaswal (2009) | United States | N = 65  Approximately half male | Most White | Most middle class | 21 3;6  22 4;5  22 5;5 |  |
| Warlaumont et al., (2024) | United States | N = 9 | Not reported | Not reported | 0;3 and 0;6 |  |
| Yu et al., (2023) | Canada | N = 192  48 undergrads (30 F)  48 mothers (48 F)  48 early childhood educators (48 F)  48 Speech Language Pathologists (48 F) | Not reported | Not reported | 19;8 undergrads  37;0 mothers  41;4 early childhood educators  34;2 Speech Language Pathologists |  |

*Studies using the SEEDLingS corpus (Bergelson, 2017)

*Note:* When both primary caregivers education attainment was reported, this table reports Caregiver 1 or Mother
