# Supplementary material for: Beyond adult speech: why research on language development should consider speech from children
Source: Child Dev Perspect. Author manuscript; Available in PMC 2026 Mar 5. (PMC12959929; doi:10.1093/cdpers/aadaf004)
Supplement: Supplementary file 1 [file NIHMS2141844-supplement-SupplementalTable.docx]

**Supplemental Table 1**

*Sociodemographic Characteristics of Studies Cited in the Article, Alphabetized by First Author’s Last Name*

| **Study** | **Country of Participants** | **Sex** | **Race/Ethnicity** | **Socioeconomic Status** | **Age** | **Ratio/Siblings** |
| --- | --- | --- | --- | --- | --- | --- |
| Ahkar et al., (2001) | United States | N = 88 Study 1: 13 F, 11 M Study 2: 16 F, 16 M Study 3: 18 F, 14 M | Not reported | Middle income | Study 1: 2;6 Study 2: 2;1 Study 3: 2;1 |  |
| Bernier & White (2019) | Canada | N = 88 Study 1: 48 Study 2: 40 | Not reported | Not reported | Study 1: 1;10 Study 2: 1;10 | Study 1: 23 had older siblings Study 2: 10 had older siblings |
| Blackstock-Bernstein (2019) | United States | N = 117 children 56 F, 61 M N = 61 teachers 61 F | Children: 94% Hispanic Teachers: 84% White | Children: 90% lived in households receiving public assistance or had incomes <130% of the federal poverty level Teachers: 39 BA, 24 Associate’s, 29 some college, 6 high school or unspecified | Children: 4;6 |  |
| Bornstein et al., (2004) | United States | N = 110 55 first-borns (26 F, 29 M) 55 second-borns (26 F, 29 M) | Not reported | Lower-middle class to upper middle class Hollingshead index: First-borns = 53.95 Second-borns = 55.11 | First-borns at 1;8 Second-borns at 1;8 | Average age gap of 2;11 |
| Bulgarelli & Bergelson (2022) | United States | N = 126 Study 1a N = 54 26 F, 28 M Study 1b N = 18 11 F, 7 M Study 2 N = 54 28 F, 26 M | Study 1a: 74% White 4% Black or African American 20% other or multiracial Study 1b: 100% White Study 2: 89% White 3% Asian 8% other or multiracial | Study 1a: 24 advanced degree 24 associate’s or bachelor’s Remaining < associate’s or bachelor’s Study 1b: 9 advanced degree 7 associate’s or bachelor’s 2 < associate’s or bachelor’s Study 2: 35 advanced degree 14 bachelor’s or associate’s 2 < bachelor’s or associate’s | 0;7-0;9 |  |
| Bulgarelli & Bergelson (2023) | United states | N = 54 34 F, 20 M | 87% White 13% Multiracial, multiethnic, other | 32 advanced degree 16 associate’s or bachelor’s 2 < associate’s or bachelor’s 4 did not report | 1;1-1;3 |  |
| *Bulgarelli & Bergelson (2024) | United States | N = 44 21 F, 23 M | 42 White 2 multiracial | 18 mothers had an associate’s or bachelor’s degree, 22 mothers had an advanced degree | Longitudinal from 0;6-1;5 |  |
| *Bulgarelli et al., (2021) | United States | N = 44 21 F, 23 M | 42 White 2 multiracial | 18 mothers had an associate’s or bachelor’s degree, 22 mothers had an advanced degree | Longitudinal from 0;6-1;5 |  |
| Bunche et al (2024) | United States, Canada, England, Argentina, Mexico, Papa New Guinea | N = 69 total 34 M | Not reported | Not reported | 1;2 (0;2-3;0) | Household size: 5.1 (2-14) |
| Casillas et al., (2020) | Mexico | N = 10 5 F, 5 M | Not reported | 2 No education 2 Preparatory 3 Primary 3 Secondary | 0;2 to 3;0 | Mean household size: 8 |
| Casillas et al., (2021) | Papua New Guinea | N = 10 5 F, 5 M | Not reported | 1 Preparatory 4 Primary 5 Secondary | 0;1 to 2;11 | Mean household size: 7 |
| ManyBabies Consortium | Around the world | Not reported | Not reported | Not reported | 0;3 – 1;3 |  |
| Cooper et al., (2018) | Canada | N = 54 | Not reported | Not reported | 2;6-3;0 |  |
| Cristia et al., (2019) | Tsimane, Bolivia | Not reported | Not reported | Forager-farmer community | 0-1;0 | Total fertility rate of 9 births, 30 month interbirth interval |
| Duncan et al., (2020) | United States | N= 44 59% M | 45% White 25% African American 18% Hispanic 11% Other | 84% received child-care subsidy (low income) | 4;9 | 2 teachers per 20-25 children |
| Fennel & Byers-Heinlein (2014) | Canada | N = 61 Bilinguals N = 30 16 F Monolingual N = 31 15 F | Not reported | Not reported | Bilinguals: 1;5 (1;4-1;6) Monolinguals: 1:5 (1;4-1;6) |  |
| Fernald et al. (1989) | French, Italian, German, Japanese, British English and American English | N = 60 30 F, 30 M | Not reported | Not reported | 0;10-1;2 |  |
| Foster (2021) | United States | N = 357 49% F, 51% M | 29% Black 24% White 42% Hispanic | Low-income | 4;6 |  |
| Galindo & Montag (under review) | United States | N = 26 15 F, 11 M | Not reported | Not reported | 0;6-1;0 | N=13 with older siblings |
| Graf Estes & Hurley (2013) | United States | N = 82 Study 1 N = 28 13 F Study 2 N = 26 13 F, 13 M Study 3 N = 28 14 F | Not reported | Not reported | 1;5.5 (1;5-1;6) |  |
| Greenwood et al., (2011) | United States | N = 30 | 77% European American 10% African American 7% Hispanic Remainder Asian, Pacific-Islander, multi-race | Middle to Upper SES | Longitudinal, 1;3 at start |  |
| Gurgand et al., (2023) | France | N = 12296 49% F | Not reported | Average education: 3.85 (3 = high school diploma, 4 = 2 years after high school diploma) | 2;0 | 5457 only children 4683 had 1 sibling 1727 had 2 siblings 429 > 2 siblings |
| Hart & Risley (1995) | United States | N = 42 | Not reported | Low to high SES | Longitudinal, 0;7-3;0 |  |
| Havron et al., (2019) | France | N = 1276 | Not reported | Not reported | 2;0, 3;0, 5;0-6;0 | 43% had older sibling Average age gap = 3;8 |
| Havron et al., (2022) | Singapore | N = 677 47.71% F | 61% Chinese 20% Malay 19% Indian | Not reported | 2;0, 4;0, 4;6 |  |
| Henry & Rickman (2007) | United States | N = 630 52.8% M | 50.4% White 38% African American 11.6% Other | 24% less than high school education 27.2% high school diploma 48.8% more than high school | 4 year olds | 39% had an older sibling Average age gap = 4;1 |
| Hippe & Ferjan Ramirez (2022) | United States | N = 24 12 M | Not reported | Hollingshead Index = 48.8 Range of SES | Longitudinal, 0;6, 0;10, 1;2, 1;6, 2;0 | 38% had older sibling |
| Howard et al., (2014) | United States | N = 82 Between Subjects N = 50 27 F, 23 M Within Subjects N = 32 18 F, 14 M | Not reported | Between Subjects: Mean Family Income $91k Within Subjects: Mean Family Income $71k | 1;7 |  |
| Hustad et al., (2021) | United States | N = 538 281 F, 257 M | 471 White 7 Black 9 Asian 1 American Indian 1 Native Hawaiian/Pacific Islander 31 More than 1 race | 2-Factor Hollinghead Social Index Mean = 55.55 | 2;6 to 10;0 |  |
| Huttenlocher et al., (2010) | United States | N = 47 | 9 African American 3 Asian 5 Hispanic 30 White | 5 High School only 10 Some college 16 Bachelor’s degree 16 Advanced degree | Longitudinal, 1;2 to 3;10 | 29 first-borns 18 later-borns |
| Immel & Liberman (2024) | United States | N = 96 53 F, 43 M | 64 White 15 Multiracial 10 Hispanic/Latino 2 Asian 1 White | 87.5% had one or more parents with a college degree | 0;9 (0;8-1;2) |  |
| Jones & Adamson (1984) | United States | N = 32 16 F, 16 M | Not reported | Middle class | 1;8 | 16 first-borns 16 second-borns Mean age gap = 2;9 |
| Justice et al., (2022) | United States | N = 1095 49% F | 67% White 8% Black 12% Hispanic/Latino | 45% with college degree or higher |  |  |
| Kalenkovich et al., (2025)* | United States | N = 44 21 F, 23 M | 42 White 2 multiracial | 18 mothers had an associate’s or bachelor’s degree, 22 mothers had an advanced degree | Longitudinal from 0;6-1;5 |  |
| Katus et al., (2024) **Gambia** | Gambia | N = 200 No differences in sex ratio | Mandinka ethnic group | 59% no formal education 13% limited formal education 3% complete primary education 18% some secondary education 6% complete secondary education | Longitudinal, 0;5, 0;8, 1;0, 1;6, 2;0 |  |
| Katus et al., (2025) **UK** | United Kingdom | N = 60 No differences in sex ratio | Not reported | Majority received undergraduate or postgraduate education | Longitudinal, 0;5, 0;8, 1;0, 1;6, 2;0 |  |
| Kuczynski et al., 1987 | United States | N = 24 13 F, 11 M | Mothers were White | Mothers were middle class, high school or college graduates | Longitudinal, Younger: Mean 1;3 in the middle Older: Mean 2;5 in the iddle | N = 16 had younger siblings |
| *Laing & Bergelson (2024) | United States | N = 44 21 F, 23 M | 42 White 2 multiracial | 18 mothers had an associate’s or bachelor’s degree, 22 mothers had an advanced degree | Longitudinal from 0;6-1;5 | Mean age gap: 4;1 |
| Lee et al., (1999) | United States | N = 436 children 207 F, 229 M N = 56 adults 27 F, 29 M | Not reported | Not reported | 5;0-18;0 |  |
| Loukatou et al., (2022) **France** | France | N = 3 2 F, 1 M | Not reported | Not reported | Longitudinal, 1;0-3;0 | 1 had one older sister 1 had two older sisters 1 had two older brothers |
| Loukatou et al., (2022) **Africa** | Lesotho, Africa | N = 3 2 F, 1 M | Not reported | Not reported | Longitudinal, from 2;1 to 3;2 | 1 had older sibling 2 had older cousins in the household |
| Ma et al., (2011) | United States | N = 64 Study 1 N = 48 24 F Study 2 N = 16 9 F |  |  | Study 1: 1;9 (1:8-1:10) Study 2: 2;4 (2;2-2;5) |  |
| Marchman & Fernald (2008) | United States | N = 28 | Primarily Caucasian | Primarily college-educated | Longitudinal, 2;1 and 8;5 |  |
| Mashburn et al., (2009) | United States | N = 1812 924 F, 888 M | 407 African American 196 Latino 923 White 262 Other 24 Missing | Average 13.1 years of maternal education | Preschool children | Average class size 17.6 kids Average child-teacher ratio 7.67:1 |
| Okocha et al., (2024) | United States | N = 369 189 F, 177 M | 63% White 9% Black 6% Latinx 4% Asian 18% more than one | 44% annual income < 50k Range from <25k to > 200,000k | 2;0 (1;0-3;1) |  |
| Oshima-Takane & Robbins (2003) | Canada | N = 14 |  |  | 1;9 | All later-borns |
| Perry et al., (2022) | United States | N = 29 17 F, 12 M | 13 White Hispanic 1 Mutiracial Hispanic 8 White 5 Black 2 Asian | Not reported | 3;0 |  |
| Pine (1995) | United States | N = 18 8 F, 10 M | Not reported | Middle class | Longitudinal, starting when first born was 0;11 | 9 first-borns 9 second-borns Mean age gap 2;6 |
| Prime et al., (2014) | Canada | N = 385 50% M | 57% Caucasian 7% Black 27% Asian 9% other | Maternal education mean: 15.59 Household income: $65k-75k | 3;0 | 60% had 1 sibling2 40% had 2 or more |
| Rosslund et al., (2025) | Norway | N = 6163 51.4% F | Not reported | Median SES = lower-level university degree | 1;10 average Range from 0;8 to 3;0 | 59.9% had one or more older siblings (range = 0-6) 10.3% had one or more younger siblings (range = 0-2) Average age gap was 5.25 years |
| Rowe et al., (2012) | United States | N = 62 30 F, 32 M | 12 Black 35 White 6 Two or more races 9 Hispanic | Annual income: 5 Less than $15000 13 $15000-34999 8 35000-49999 12 50000-74999 10 75000-99999 14 100000 or more | Longitudinal, every 4 months from 1;2 to 3;10 |  |
| Ryalls et al., (2000) | United States | N = 30 16 F, 14 M | Majority Caucasian | Majority middle class | 1;4 (range 1;2 to 1;6) |  |
| Sachs & Devin (1976) | United States | N = 4 | Not reported | Not reported | 3;9 to 5;5 |  |
| Scaff et al., (2024) | Bolivia | N = 24 8 F, 16 M | Not reported | Not reported | 2;6 (range 0;6 to 4;10) | Average household size: 8 Average number of siblings: 2.9 |
| Schaffer & Liddell (1984) | United Kingdom | N = 16 teachers | Not reported | Not reported | Observed children were 2;7 |  |
| Schick, Daum, & Stoll (2025) | Peru, Switzerland | N = Study 1 N = 127 N=67 in Peru, 37F N = 60 in Switzerland, 29 F Study 2 N = 61 28 F | Not reported | Not reported | Study 1: 1;1 (0;8-1;8) Study 2: 1;2 (0;8-1;8) | Study 1 Peru: 3.85 siblings (range 0-10) Study 1 Switzerland: 0.47 (range 0-2) Study 2: 0.44 (range 0-4) |
| Schick & Stoll (2025) | Peru | N = 12 8 F, 4 M | Not reported | Not reported | 1;0 to 4;9 |  |
| Schwab et al., (2018) | United States | N = 41 22 F, 19 M | 100% African American fathers | Fathers’ M education: 12.5 (10-16) – high school degree | 2;0 Fathers’ M = 29y (18-52) |  |
| Shatz & Gelman (1973) | United States | N = 21 Study 1: 16 Study 2: 5 3 F, 2 M | Not reported | Middle to upper class | 4;4 (range 3;1 to 5;0) |  |
| Shneidman & Goldin-Meadow (2012) | Mexico United States | N = 9 each Mexico: 6 F, 3 M USA: 7 F, 2 M | USA: 4 Caucasian 3 African American 1 Mixed Race 1 not reported | USA: 4 < 35,000 1 between 50,000-74999 4 > 75000 | 1;2 | Average number of children present Mexico: 4.7 USA: 1.3 |
| Shneidman et al., (2013) | United States | N = 30 18 F, 12 M | Not reported | 5 graduated high school 6 some college 10 college degree 9 advanced degree | Longitudinal, every four months from 1;2 to 3;6 | 10 participants had siblings, 2.3 siblings on average |
| Shneidman et al., (2016) | United States Mexico | N = 142 Study 1: N = 76 from US Study 2: N = 66 36 Yucatec Mayan, 30 from US | Study 2: US – Primarily White | Study 1: Primarily Middle class Study 2: US Primarily Middle Class, 17.2 years of education Mayan – 5.6 years of education | Study 1: 2;6 Study 2: 2;7 | Study 2: Mayan children larger household size (2.6 siblings) than US (0.5 siblings) |
| Shutts et al., (2010) | United States | N = 64 22 F | 100% White | Not reported | 3;5 |  |
| Simonsen et al., (2014) | Norway | N = 6574 reports 51% F, 49% M | Not reported | 3% Basic education (<9 years) 30% Short further education (12 years) 42% Medium further education (14-16 years) 24% Long further education (>16 years) | 0;8 to 3;0 |  |
| Sparks et al., (2024) | United States | N = 26 50% male/female | 7.29% African American/Black 19.2% Asian American/Pacific Islander 38.5% Caucasian/White 7.69% Hispanic/Latinx 30.8% multiracial 3.85% other | Not reported | 2;11-5;11, Mean = 4;6 |  |
| Tingley & Allen (1975) | United States | N = 20 | Not reported | Not reported | 5;0-11;5 |  |
| Van Heugten & Johnson (2017) | Canada | N = 80 Study 1 N = 40 21 M Study 2 N = 40 23 M | Not reported | Study 1: 5.7* Study 2a:6.1 Study 2b: 5.6 *measured on scale of 1 (some high school) to 7 (postgraduate degree) | Study 1: 1;0 Study 2a: 1;2 Study 2b: 1;6 |  |
| VanderBorght & Jaswal (2009) | United States | N = 65 Approximately half male | Most White | Most middle class | 21 3;6 22 4;5 22 5;5 |  |
| Warlaumont et al., (2024) | United States | N = 9 | Not reported | Not reported | 0;3 and 0;6 |  |
| Yu et al., (2023) | Canada | N = 192 48 undergrads (30 F) 48 mothers (48 F) 48 early childhood educators (48 F) 48 Speech Language Pathologists (48 F) | Not reported | Not reported | 19;8 undergrads 37;0 mothers 41;4 early childhood educators 34;2 Speech Language Pathologists |  |

*Studies using the SEEDLingS corpus (Bergelson, 2017)

Notes:

When both primary caregivers education attainment was reported, this table reports Caregiver 1 or Mother
